# Absence of nuclease activity in commonly used oxygen-scavenging systems

**DOI:** 10.1186/s13104-017-2929-6

**Published:** 2017-11-21

**Authors:** Hailey L. Gahlon, Paul Poudevigne-Durance, David Rueda

**Affiliations:** 1 Molecular Virology, Department of Medicine, Imperial College London, Du Cane Road, London, W12 0NN UK; 2 Single Molecule Imaging Group, MRC London Institute of Medical Sciences, Du Cane Road, London, W12 0NN UK

**Keywords:** Single-molecule imaging, Oxygen scavenging system, Protocatechuate-3,4-dioxygenase, Protocatechuic acid, Glucose oxidase, Catalase

## Abstract

**Objective:**

Oxygen scavenging systems are routinely used during single-molecule imaging experiments to improve fluorescent dye stability. Previous work has shown nuclease contamination in the commonly used oxygen scavenging systems. This study evaluates the potential for nuclease contamination in these oxygen scavenging systems.

**Results:**

Linear and plasmid DNA was incubated with two different oxygen scavenging systems (1) protocatechuic acid (PCA)-protocatechuate-3,4-dioxygenase (PCD) and (2) glucose-coupled glucose oxidase/catalase (GODCAT). No nucleic acid degradation was observed on single and double-stranded linear DNA and plasmid DNA, indicating the absence of nuclease contamination in these oxygen scavenging systems.

## Introduction

Previous work has suggested that contaminant nuclease enzymes could be affecting single-molecule studies [1]. Using size-exclusion chromatography, this study reported the presence of nuclease contamination in the 40- and 100 kDa elution fractions of routinely used OSS system with protocatechuic acid (PCA)-protocatechuate-3,4-dioxygenase (PCD), with the 40 kDa fraction exhibiting the highest nuclease activity. Such contamination could compromise DNA integrity during a single-molecule experiment, leading to false or inaccurate data interpretation. Therefore, we decided to test if we can observe similar DNA degradation on linear single- and double-stranded DNA constructs as well as circular plasmid DNA in the presence of the commonly used OSS systems, (PCA)-(PCD) and glucose-coupled glucose oxidase/catalase (GODCAT).

## Main text

### Methods

#### Linear DNA substrates

Linear DNA sequences include the 92-mer strand, 5′-CCC TAC ATC CAT TCC TCG CGT TTT TT(Cy3-T) (T)_65_-3′, the single-stranded 66-mer, 5′-Cy3 ACC TCC TCA CCT TTC CTC TTT GCT TTC CCC TTT TCT ACA ATA CGG ATA CGG ACG GCT GCA TCT CTG-3′ and the 66-mer double-stranded DNA that contains a complementary 66-mer sequence annealed to the above mentioned 66-mer sequence with a 5′ biotin. DNA oligonucleotides were purchased from Eurofins Genomics.

#### Plasmid DNA substrates

Double-stranded circular pUC19 plasmid DNA (2686 bp) was tested for potential nuclease contamination. Plasmid DNA was purchased from Invitrogen.

#### Nuclease assay

**Fig. 1:**
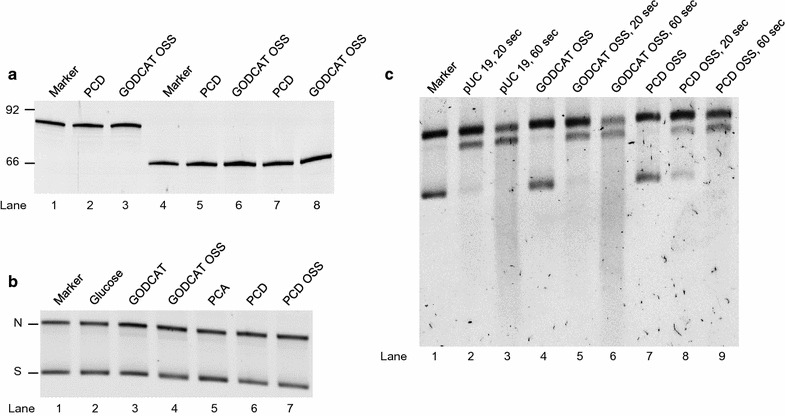
Analysis for potential nuclease contamination in commonly used single-molecule oxygen scavenging systems. The Marker lanes have DNA only, no protein was added. **a** Single-stranded DNA 92-mer (lanes 1–3) and 66-mer (lanes 4–6) and double-stranded 66-mer duplex (lanes 7–8) were tested. **b** Double-stranded circular pUC19 plasmid DNA (2686 bp), the faster migrating band is supercoiled DNA (S) and the slower migrating band is nicked DNA (N). **c** pUC19 plasmid DNA treated with DNase1 for indicated times. Lane 1, puC19 plasmid marker; Lanes 2 and 3, pUC19 treated with DNase1; Lane 4, pUC19 plus GODCAT OSS; Lanes 5 and 6, pUC19 plus GODCAT OSS treated with DNase1; Lane 7, pUC19 plus PCD OSS; Lanes 8 and 9, pUC19 plus PCD OSS treated with DNase1

Each reaction contained 0.01 nmol of Cy3 DNA, except for the double-stranded DNA reactions (Fig. 1a, lanes 7–8) that contained 0.01 nmol of Cy3 DNA and 0.01 nmol of the complementary DNA strand. Concentrations for each specified reaction are as follows, 60 nM protocatechuate-3,4-dioxygenase (PCD) (accounting for the 40% of PCD with stabilizer), 5 mM PCA, 35 units/mL glucose oxidase (GOD), 62,500 units/mL catalase (CAT) and 1% d-Glucose. GODCAT OSS includes glucose oxidase, catalase and d-Glucose while PCD OSS contains both PCD and PCA. For experiments with DNase1 (Turbo DNase Ambion, Life Technologies), reactions were performed at a final concentration of 0.4 U in 100 μL and stopped at 20 and 60 s. Reactions were tested in duplicate with the same buffer conditions as Senavirathne et al. [1]. containing 50 mM Tris–HCl pH 7.5, 100 mM NaCl, 5 mM MgCl_2_ and 0.1 mM DTT at 22 °C for 40 min. Following each reaction, linear DNA was separated on a 15% polyacrylamide gel and plasmid DNA on a 1% agarose gel. SYBR gold was used for nucleic acid staining in addition to direct visualization with DNA containing Cy3. Gels were visualized on a FUJIFILM FLA-5100 instrument.

## Results and discussion

Linear, single- and double-stranded, and plasmid DNA substrates were tested for nuclease contamination in oxygen scavenging systems that are routinely used during single-molecule imaging. The nuclease assay consisted of incubating the OSS with the respective DNA and running on a polyacrylamide, in the case of linear DNA, and an agarose gel in the case of plasmid DNA. Using standard protocols [2–4], we did not observe any DNA degradation on all DNA substrates tested under the same buffer conditions used by Senavirathne et al. [1] Fig. 1.

We have compared our reagent sources to those in the previous manuscript by Senavirathne et al. Most of the reagents come from the same source and lot number (Table 1), suggesting that potential contamination from the commercial source may not provide an answer for the degradation observed previously by Senavirathne et al.

**Table 1:** List of materials and providers used in this work

Product	Senavirathne et al.			Gahlon et al.		
	Supplier	Cat. no.	Lot no.	Supplier	Cat. no.	Lot no.
PCD	Sigma	P8279-25UN	SLBL 454 IV	Sigma	P8279-25UN	SLBL 454 IV
PCA	NP^a^			Sigma	37580-100G-F	BCBH2769V
GOD	Sigma	G2133-10KV	BCBP8730V	Sigma	G2133-10KV	BCBP8730V
Catalase	Sigma	C9322-1G	SLBG1321V	Sigma^b^	C100-50MG	SLBH8572V
Glucose	NP^a^			Sigma	G8270-5KG	SZBE0450V

^a^Reagent source not specified by Senavirathne et al.

^b^We used the same provider, but purchased in lower quantities (50 mg vs. 1 g)

Additionally, we have compared our experimental conditions to those in the manuscript by Senavirathne et al. (Table 2). While most concentrations used are similar, there are a few exceptions, which are outlined in Table 2. It is important to note that the largest amount of degradation observed by Senavirathne et al. was for the catalase enzyme. For this, we used a larger concentration of catalase in comparison to the previous study and still did not observe any nucleic acid degradation in our assays.

**Table 2:** List of reagents used in this work

Reagent	Senavirathne et al.	Gahlon et al.
Buffer	50 mM Tris–HCl	50 mM Tris–HCl
pH	7.5	7.5
NaCl (mM)	100	100
MgCl_2_ (mM)	5	5
DTT (mM)	0.1	0.1
Incubation time (min)	40	40
Temperature (°C)	22	22
PCA (mM)	5	5
PCD	0.21 U/mL	60 nM^a^
Glucose, (%)	0.8	1
GOD (U/mL)	165	35
Catalase (U/mL)	2170	62,500
Linear DNA	10 ng/µL^b^	100 nM
Plasmid (ng/µL)	10	10

^a^We used conditions comparable to their highest concentration

^b^Concentration varies from ~ 350 to 600 nM depending on construct size

These findings are important to rule out the potential for nuclease contamination in the common oxygen scavenging systems used in single-molecule imaging [1]. We feel it is of value to reassure the wider scientific community that we do not observe nucleic acid degradation with our samples and that alternative explanations may account for the previously reported DNA degradation [1]. For example, while the most significant degradation observed in the previous work [1] was for the pBS SK (-) plasmid, in our hands we did not observe any degradation with the pUC19 plasmid DNA (Fig. 1b). This discrepancy could be due to a restriction enzyme contamination rather than a nuclease, in this instance the observed degradation would be sequence specific. If this is true, experiments involving plasmid DNA or sequences with restriction sites may require prior removal of any contaminant enzymes in the oxygen scavenging systems. Alternatively, the nuclease contamination could be an isolated set of data either from contamination introduced during the preparation of the enzyme-based oxygen scavenging samples or contamination directly from the commercial sources. However, the PCD and GOD enzymes tested by Senavirathne et al. and by us were from the same supplier with the same lot number (Table 1), suggesting that that commercial source may not provide a complete answer. Therefore, we encourage labs utilizing oxygen-scavenging systems to test for contaminants that might degrade DNA or RNA in their own experiments to mitigate any experimental artifacts.

## Limitations

Further DNA substrates should be tested with both oxygen scavenging systems (i.e. glucose oxidase/catalase and the PCA PCD system). It would also be good to test in a systematic way other nucleic acid substrates to asses if DNA sequence may be important.

## Abbreviations

OSS: oxygen scavenging system
PCD: protocatechuate-3,4-dioxygenase
PCA: protocatechuic acid
GODCAT: glucose-coupled glucose oxidase/catalase
